# Investigating the utilization mechanism and kinetics of sialic acid mimetics in mammalian cell lines

**DOI:** 10.1039/d5cb00193e

**Published:** 2025-10-29

**Authors:** Eline A. Visser, Daniël L. A. H. Hornikx, Moritz Rahm, Özden Öztürk, Venetia Psomiadou, Matteo Calzari, Celine Mennen, Sam J. Moons, Martin Jaeger, Dirk J. Lefeber, Christian Büll, Thomas J. Boltje

**Affiliations:** a Department of Synthetic Organic Chemistry, Institute for Molecules and Materials, Radboud University Nijmegen Toernooiveld 1 6525 ED Nijmegen The Netherlands thomas.boltje@ru.nl; b Department of Biomolecular Chemistry, Institute for Molecules and Materials, Radboud University Nijmegen Heyendaalseweg 135 6525 AJ Nijmegen The Netherlands christian.bull@ru.nl; c Department of Laboratory Medicine, Translational Metabolic Laboratory, Radboud University Medical Center Geert Grooteplein Zuid 10 6525 GA Nijmegen The Netherlands; d Synvenio B.V. Toernooiveld 1 6525 ED Nijmegen The Netherlands; e Department of Experimental Internal Medicine, Radboud Institute for Medical Innovations, Radboud University Medical Center Geert Grooteplein Zuid 10 6525 GA Nijmegen The Netherlands; f Department of Neurology, Donders Institute for Brain, Cognition, and Behavior, Radboud University Medical Center Geert Grooteplein Zuid 10 6525 GA Nijmegen The Netherlands

## Abstract

Sialic acid mimetics (SAMs) are chemically modified derivatives of sialic acids that can act as metabolic inhibitors or as sugar donors for sialyltransferases. This makes SAMs highly useful research tools to study and manipulate the biosynthesis of sialic acid-carrying glycans (sialoglycans). Moreover, SAMs that inhibit aberrant sialylation in cancer cells are emerging as potential therapeutics. Despite the wide use of SAMs, many aspects regarding their cellular uptake and metabolic fate are unknown. Here, we investigated the metabolic fate of an inhibitory SAM (P-SiaFNEtoc) and an incorporative SAM (P-SiaNPoc) in various mammalian cell lines. Using kinetic experiments and read-outs based on sialic acid-binding lectins, click chemistry, and nucleotide sugar analysis, we monitored the key steps of cellular SAM utilization. We found differences in the metabolism of SAMs that determine their potency in different mammalian cell lines. By identifying a murine macrophage cell line that is insensitive to SAMs, we have identified esterase activity as a bottleneck for the cellular utilization of SAMs. This study contributes to the understanding of the mechanisms underlying SAMs utilization in mammalian cell lines and provide relevant considerations for the future chemical design of SAMs and their application in mammalian systems.

## Abstract 2

Sialic acid mimetics (SAMs) are chemically modified sialic acid derivatives that can act as metabolic inhibitors or as sugar donors for sialyltransferases.

## Graphical abstract

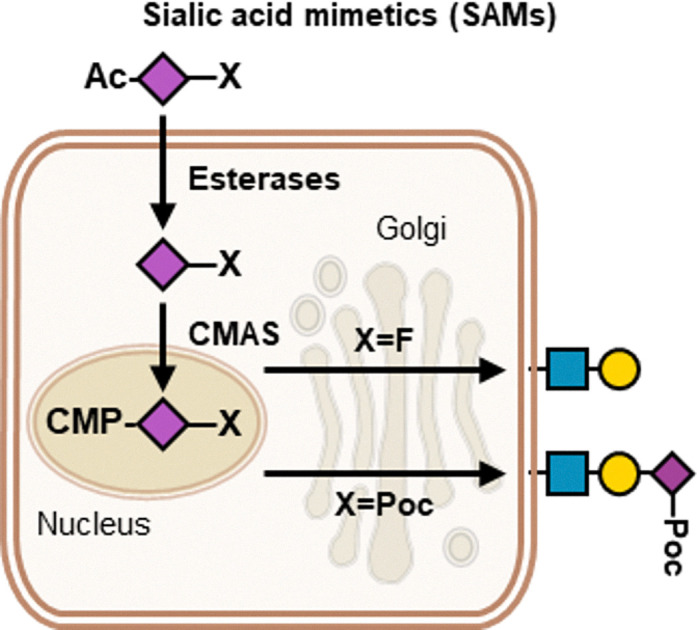

## Introduction

Mammalian glycans are capped by sialic acids (Sias), a family of negatively charged nine-carbon monosaccharides. The most common Sia in mammals is *N*-acetylneuraminic acid (Neu5Ac), followed by *N*-glycolylneuraminic acid (Neu5Gc) which is not biosynthesized in humans.^1,2^ Due to their terminal position on glycans, Sias are at the center of many molecular interactions. They regulate the stability of proteins and membranes, mask underlying monosaccharides such as galactose from recognition, and they form the ligands for immune modulatory sialic-acid-binding immunoglobulin-like lectin (Siglec) receptors.^3–6^ Moreover, several pathogens engage sialic acids to bind and infect host cells.^7^ For example, influenza viruses use sialic acids on epithelial cells to infect the airways.^8^ The aberrant expression of sialic acids is frequently observed in cancers and contributes to immune evasion and metastasis.^9–11^ Therefore, sialic acids form potential therapeutic targets in infection, immunity, and cancer.

**Scheme 1:**
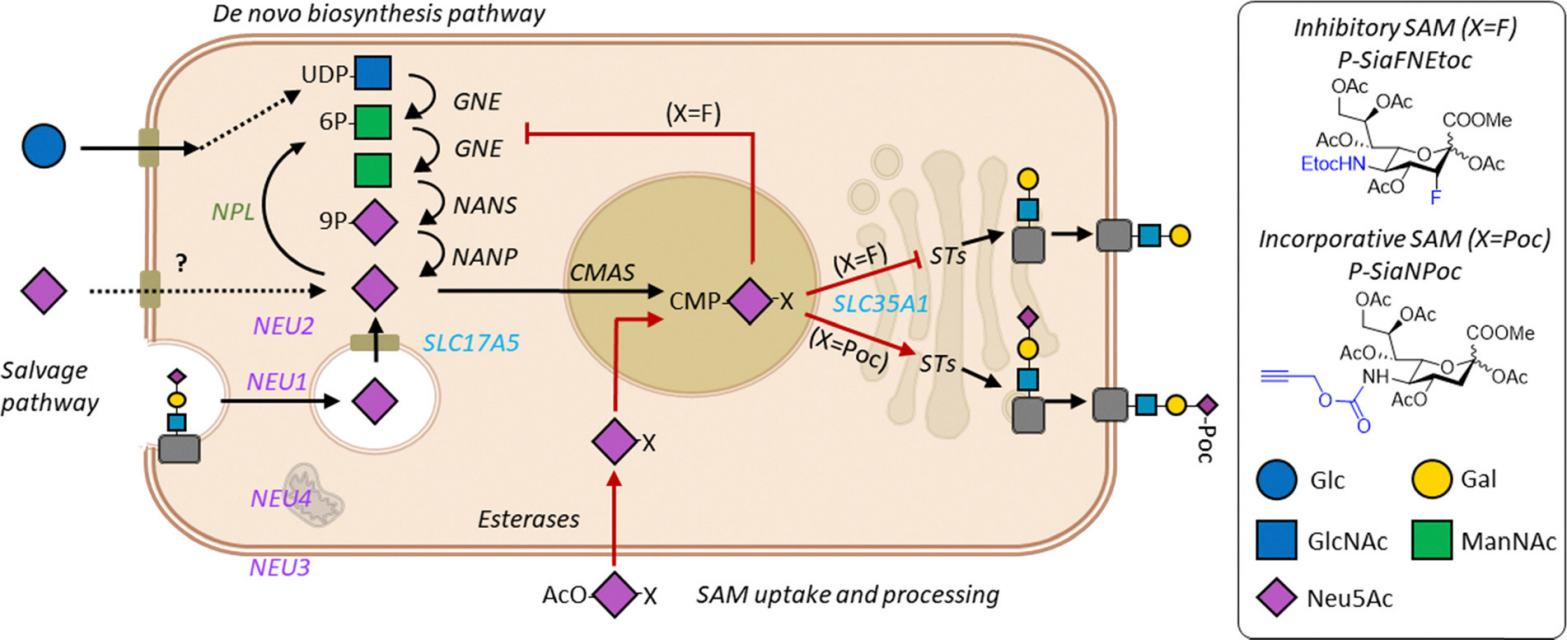
Sialic acid biosynthesis and utilization of SAMs. Sialic acids (Sia) are formed in the *de novo* biosynthesis pathway from metabolism of glucose (Glc).^6,17^ Glc is converted to UDP-*N*-acetylglucosamine (GlcNAc) followed by the two-step action of GNE (UDP-*N*-acetylglucosamine 2-epimerase/*N*-acetylmannosamine kinase) that yields *N*-acetyl-d-mannosamine (ManNAc) 6-phosphate and ManNAc. ManNAc is converted by the sialic acid synthase NANS to Sia-9-phosphate followed by conversion to Sia by NANP (*N*-acylneuraminate-9-phosphatase). Exogenous free Sias and SAMs, or recycled Sias cleaved by neuraminidases (NEU1-4), can directly enter the cytoplasm or are exported by SLC17A5 (Sialin) from the lysosome in the salvage pathway. SAMs protected with acetyl groups (OAc) are deacetylated by esterases and free Sias in the cytoplasm are activated in the nucleus by conjugation to cytidine monophosphate (CMP) by cytidine monophosphate *N*-acetylneuraminic acid synthetase (CMAS). CMP-Sia is transported to the Golgi apparatus *via* SLC35A1 and serves as the donor substrate for the sialyltransferases (STs). SAMs with a fluorine group (X = F) inhibit sialylation resulting in uncapped glycans. Propargyloxycarbonyl (X = Poc) modified SAMs are incorporated into sialoglycans.^37^ Glycan symbols are drawn according to the SNFG nomenclature.^38^

Sias in mammalian cells derive either from the *de novo* biosynthesis or the salvage pathway. During *de novo* biosynthesis, Sias are generated from the precursor sugar *N*-acetylmannosamine (ManNAc) (Scheme 1). ManNAc is converted in the cytoplasm to Sia by the sequential action of the three enzymes GNE, NANS, and NANP.^12^ Cytosolic Sia then enters the nucleus and the anomeric center is activated by conjugation to cytidine monophosphate (CMP) by the cytidine monophosphate *N*-acetylneuraminic acid synthetase (CMAS) enzyme.^13^ The resulting CMP-Sia sugar donor is transported into the Golgi *via* the SLC35A1 transporter.^14^ In the salvage pathway, Sia is obtained from the internalization and recycling of membrane sialoglycans or from indirect uptake of free (dietary) Sias *via* pinocytosis. Recycling sialoglycans enter the lysosome where they are degraded by sialidases (NEU) and the released Sia is transported into the cytosol by the transporter SLC17A5 (sialin).^15^ A cytoplasmic aldolase NPL can catabolize Sia into ManNAc and pyruvate or salvaged Sias are CMP activated to regenerate CMP-Sia which is transported to the Golgi.^16^ In the Golgi, CMP-Sias are the donor substrate for the twenty sialyltransferase isoenzymes (STs) that cap glycans with Sia(s). STs can conjugate Sias either by α2-3 (ST3Gal1-6), α2-6 (ST6Gal 1/2 and ST6GalNAc1-6), or α2-8 (ST8Sia1-6) linkages to galactose (Gal), *N*-acetyl galactosamine (GalNAc) and *N*-acetylneuraminic acid (Neu5Ac) containing glycoconjugates, respectively. Mature sialoglycoproteins and glycolipids traffic to the cell membrane or are secreted. Although the general mammalian sialic acid biosynthesis pathway has been mapped, many aspects of Sia metabolism are not fully understood. For example, our understanding of Sia biosynthesis and recycling regulation at the enzyme and metabolic level is incomplete.

The dynamics of Sia biosynthesis can be interrogated using chemically modified monosaccharides. Chemically modified ManNAc or Sias are sugar mimetics that can be introduced into the cellular sialoglycan biosynthesis pathway *via* the *de novo* or salvage pathway, respectively.^17–19^ Exogenous ManNAc and Sias are not efficiently taken up by mammalian cells as there is no active uptake mechanism and there hydrophilic limits passive diffusion. Thus, high micromolar to millimolar concentrations are needed to achieve passive uptake of the unnatural sugars, likely *via* pinocytosis.^20,21^ Alternatively, unnatural ManNAc and Sia sugars can be acetylated (protected) to facilitate passive diffusion over the cell membrane resulting in effective concentrations in the low/medium micromolar range.^22^ Inside the cell, cytosolic esterases remove the acetyl esters and the deprotected sugar mimetic can be utilized.^23,24^ While ManNAc derivatives need to be converted to Sia by the three enzymes GNE, NANS, and NANP, Sia derivatives are directly available for CMP activation. This is reflected by the generally lower potency of ManNAc mimetics compared to Sia mimetics (SAMs).^17,25^ SAMs can be decorated with groups that enable metabolic labeling of sialoglycans for imaging, alteration of their receptor binding, and substrate-based inhibition of enzymes involved in the sialoglycan biosynthesis.^19,26–28^ For example, C-3 fluorinated SAMs such as P-3F_ax_Neu5Ac act as global inhibitors of sialylation by directly inhibiting sialyltransferases and inducing feedback inhibition of GNE, a crucial enzyme within the sialic acid *de novo* biosynthesis pathway.^28^ We have shown that introducing a ethyloxycarbony (Etoc) group to the C-5 position of this fluorinated sialic acid (P-SiaFNEtoc), the potency of sialylation inhibition was improved.^29^ We and others have shown that SAMs that inhibit sialylation can potentially be applied as cancer therapeutics in mouse models to increase immune recognition of tumors and to reduce metastasis.^30–33^ Alternatively, SAMs that carry bioorthogonal a chemical reporter (*e.g.* azide or alkyne) can be incorporated into sialoglycans, enabling their functionalization using bioorthogonal chemistry. By reacting fluorophores to SAMs, cell surface sialoglycans can be imaged in live organisms.^34^ Reaction of incorporated SAMs with small molecules can also be used to change the charge and biophysical property of the cell surface and modulate the affinity of sialic acid–Siglec interactions.^27,35^ Thus, inhibitory and incorporative SAMs are highly useful tools to study the biological functions of sialic acids and have therapeutic application potential in cancer and immunity.^36^ However, the wider application of SAMs in research particularly in biomedicine requires a better understanding of their metabolic processing and fate. Herein, we dissected the different steps of cellular utilization of an inhibitory (P-SiaFNEtoc) and an incorporative (P-SiaNPoc) SAM including their uptake, activation, incorporation, and turnover. We found that the kinetics of these SAMs largely follows the cellular sialic acid biosynthesis and turnover rates in different mammalian cell lines. Moreover, we identified esterase activity as a bottleneck for effective SAM utilization by cells. Thus, these findings provide relevant insights to further develop and apply SAMs in mammalian cells.

## Results and discussion

### Different utilization of SAMs by mammalian cell lines

**Fig. 1:**
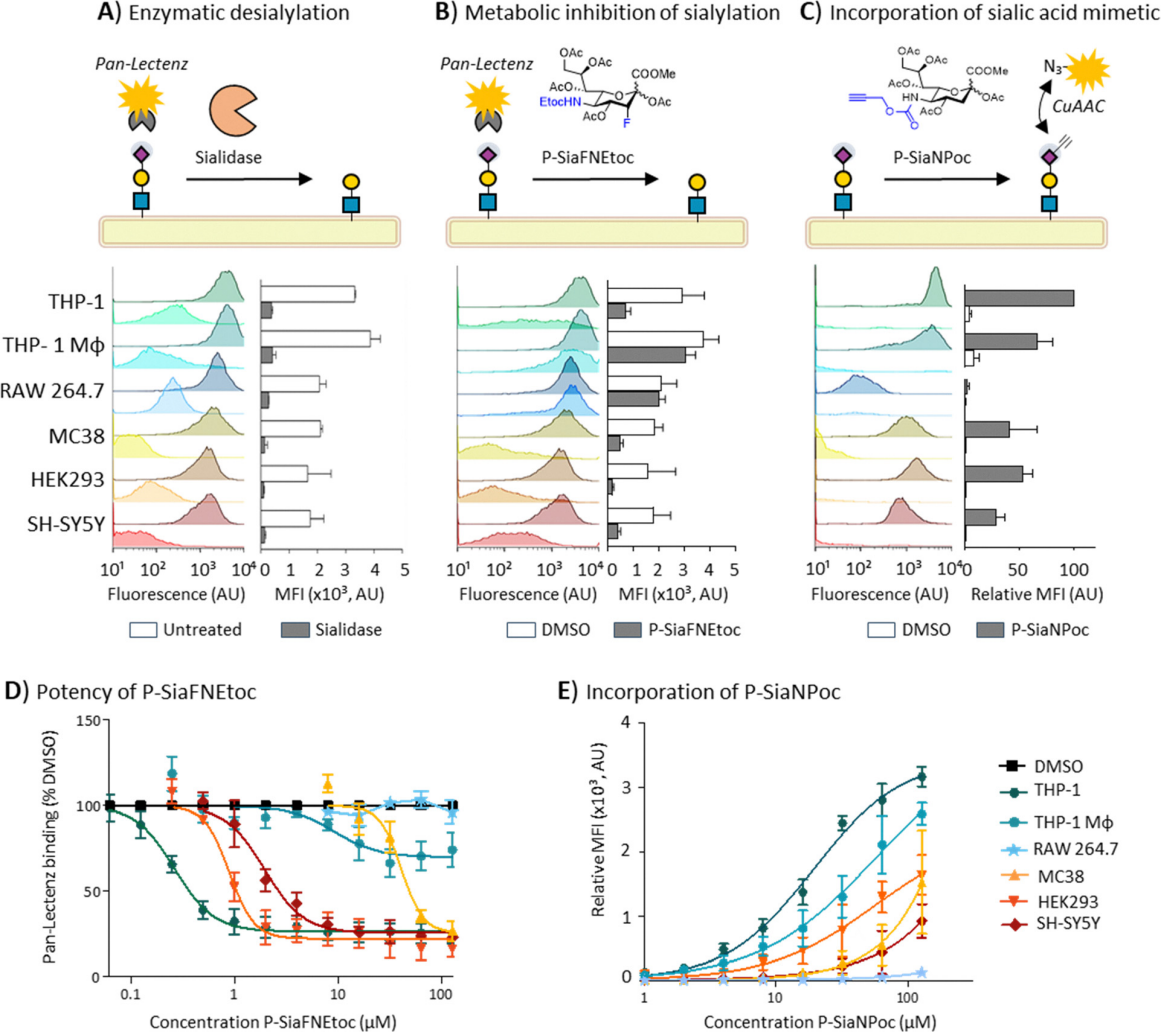
Endogenous surface sialoglycan levels and efficacy of SAMs in the mammalian cell lines THP-1, THP-1 Mφ, RAW 264.7, MC38, HEK293, and SH-SY5Y. (A) Surface sialoglycan levels of untreated cells and cells treated with 100 mU ml^−1^*C. perfringens* sialidase were measured with fluorescent Pan-Lectenz and flow cytometry analysis. Left: Representative histograms are shown. Right: bar diagrams show the corresponding average mean fluorescence intensity (MFI) values ±SD (*n* = 3). (B) Cell lines were incubated with 128 μM P-SiaFNEtoc for 72 hours or DMSO control. Left: Representative histograms show Pan-Lectenz binding to the control and treated cells. Right: bar diagram shows Pan-Lectenz binding as average MFI values ±SD (*n* = 3). (C) Cells were incubated for 72 hours with 128 μM P-SiaNPoc or DMSO control. Presence of Poc groups in surface sialoglycans was determined using CuAAC-reaction to biotin followed by detection with fluorescent streptavidin and detection by flow cytometry. Left: Histograms show cell surface labeling of sialoglycans. Right: relative MFIs were calculated by normalization to the background signal of DMSO-treated cells. The bar diagram shows the average relative MFI ±SD (*n* = 3). (D) Dose–response curves of the inhibitory P-SiaFNEtoc for the mammalian cell panel. Cells were cultured for 72 hours with increasing concentrations of P-SiaFNEtoc followed by detection of surface sialoglycans using Pan-Lectenz. Diagram shows the average percentage of Pan-Lectenz binding normalized to control DMSO-treated cells ±SEM (*n* = 3). (E) Dose–response curves for cells cultured for 72 hours with increasing concentrations of P-SiaNPoc. Diagram shows the average relative MFI normalized to DMSO-treated cells ±SEM (*n* = 3).

To study the commonalities and differences of SAM utilization between mammalian cell lines, we assessed the potency of P-SiaFNEtoc and P-SiaNPoc in a panel of human and mouse cell lines. The cell panel consisted of the human cell lines THP-1 (monocytic), THP-1 Mφ (THP-1-derived macrophages), HEK293 (embryonic kidney) and SH-SY5Y (neuroblastoma). In addition, we tested the murine cell lines RAW 264.7 (macrophage) and MC38 (colon adenocarcinoma). First, we determined the relative levels of sialoglycans present on the surface of these cells using staining with Pan-specific Lectenz reagent (Pan-Lectenz), an inactivated sialidase probe that recognizes most types of sialoglycans, followed by detection by flow cytometry analysis. We found that the cell surface sialoglycan levels differ between the cell lines with THP-1 and THP-1 Mφ cells showing the highest Pan-Lectenz binding and HEK293 and SH-SY5Y displaying the lowest sialoglycan levels. Sialidase treatment abrogated Pan-Lectenz binding almost completely (Fig. 1A). The residual signal (5–15%) is probably the result of incomplete sialidase activity or non-specific binding of the Pan-Lectenz reagent. Next, we treated the cell panel with increasing concentrations of the inhibitory SAM P-SiaFNEtoc to block the biosynthesis of sialoglycans and measured sialylation levels after 3 days. Previous studies have shown that the maximum reduction of sialylation is observed after 3 days of culture due to the time it takes for sialoglycan turnover.^29^ P-SiaFNEtoc effectively reduced sialylation in a dose-dependent manner in THP-1, HEK293, SH-SY5Y, and MC38 cells (Fig. 1B and D). THP-1 cells were most sensitive and showed maximum inhibition (EC_90_) at concentrations of 1 μM, followed by HEK293 (1.8 μM), SH-SY5Y (7.5 μM), and MC38 (83 μM) (Table 1). In the THP-1 Mφ cell, only a 30–40% reduction in Pan-Lectenz binding was observed at the highest concentration of 128 μM and sialylation of RAW 264.7 cells was barely affected by SAM treatment (Fig. 1B and D). Treatment with 200 μM and a longer incubation time of 6 days did not result in a further decrease of sialoglycan expression (Fig. S1). Additional analysis of eight more cell lines showed potent reduction of sialylation with EC_90_ values in the range of 0.7 μM to 13.5 μM (Table 1 and Fig. S2). This suggests that sialylation can be effectively inhibited in most cell lines with P-SiaFNEtoc, but that human and mouse macrophage(-like) cells respond poorly to this SAM.

**Table 1:** EC_50_ and EC_90_ values in μM for inhibition of sialylation^a^

Cell line	P-SiaFNEtoc EC_50_	Standard error log EC_50_	P-SiaFNEtoc EC_90_	Standard error log EC_90_
A549	2.20	0.042	4.01	0.109
H1299	0.35	0.017	0.71	0.036
MDA-MB-231	0.96	0.027	1.84	0.068
SKOV-3	1.10	0.041	2.33	0.098
IGROV-1	0.73	0.036	2.08	0.082
OVCAR-3	0.58	0.146	3.53	0.358
PANC-1	0.80	0.084	3.02	0.203
Capan-1	6.60	0.068	13.47	0.150
THP-1	0.27	0.044	1.08	0.127
RAW 264.7	N.I.	N.I.	N.I.	N.I.
MC-38	41.30	0.066	83.31	0.152
HEK293	0.90	0.044	1.78	0.106
SH-SY5Y	1.96	0.052	7.45	0.141

a Mammalian cell lines were cultured for 3 days with 0–128 μM (two fold dilutions) P-SiaFNEtoc or DMSO control. Pan-Lectenz binding to the cells was detected by flow cytometry. The EC_50_ and EC_90_ values were determined as the concentration where a 50% or a 90%, respectively, decrease in Pan-Lectenz binding compared to control was observed, with their corresponding standard errors of the log EC_50_ and log EC_90_ values. N.I. = no inhibition detected.

Similar effects were observed for the incorporation of P-SiaNPoc into the cell lines. This incorporative SAM results in the cell surface expression of propargyloxycarbonyl (Poc) groups that be conjugation to a fluorescent group using a bioorthogonal copper(i)-catalyzed azide–alkyne cycloaddition (CuAAC) reaction (Fig. 1C). In general, the level of signal from P-SiaNPoc incorporation correlated with the intensity of Pan-Lectenz staining with THP-1 cells showing the highest signal (Fig. 1C and E). The THP-1 Mφ showed significant incorporation of P-SiaNPoc, thus seem to be more responsive to the incorporative SAM compared to the inhibitory one, however RAW 264.7 cells showed no incorporation. To investigate if low sensitivity or resistance to the SAMs is a general feature of macrophages, we treated macrophages derived from peripheral blood monocytes with the SAMs. The primary macrophages showed incorporation of P-SiaNPoc, but were less sensitive to sialylation inhibition with P-SiaFNEtoc (20–60% inhibition) (Fig. S3). These results show that most mammalian cell lines utilize SAMs in line with the reported application of SAMs in cell lines and mice.^21,30,33,39^ Differences in effective concentrations between cell lines are likely caused by variances in (passive) uptake, the expression of transporters and enzymes involved in sialoglycan biosynthesis (*e.g.* CMAS, SLC35A1, and STs), differences in the metabolic flux, sialoglycan turnover rate, clearance rate of SAMs, and endogenous sialic acid concentrations. Others have reported that the differentiation of human peripheral blood monocytes or THP-1 monocytes into macrophages induces changes in sialyltransferase and sialidase expression levels and cell surface glycosylation.^40,41^ Accordingly, we found different expression levels of sialyltransferases (Table S1). Although it is difficult to predict glycosylation based on gene expression, altered sialyltransferase and sialidase expression and the subsequent differences in the display of sialic acids on different glycoconjugate, glycoprotein, and glycolipid repertoires and effects on the turnover of cell surface sialoglycans arguably influences the sensitivity and response of cells to P-SiaFNEtoc. Further research is needed to understand how these effect the responsiveness of cells to SAMs. In this study, we only investigated one inhibitory and one incorporative SAM, but differences can also exist between different SAMs. For example, we and others found that the use of different chemical reporters, *e.g.* alkyne or azide groups, can cause a difference in labelling efficiency in the same cell line.^25,42,43^ Additionally, the position of the modification on the nine-carbon sialic acid backbone can affect the metabolic processing efficiency by the sialoglycan biosynthesis enzymes.^44^

### Cellular incorporation kinetics of SAMs

**Fig. 2:**
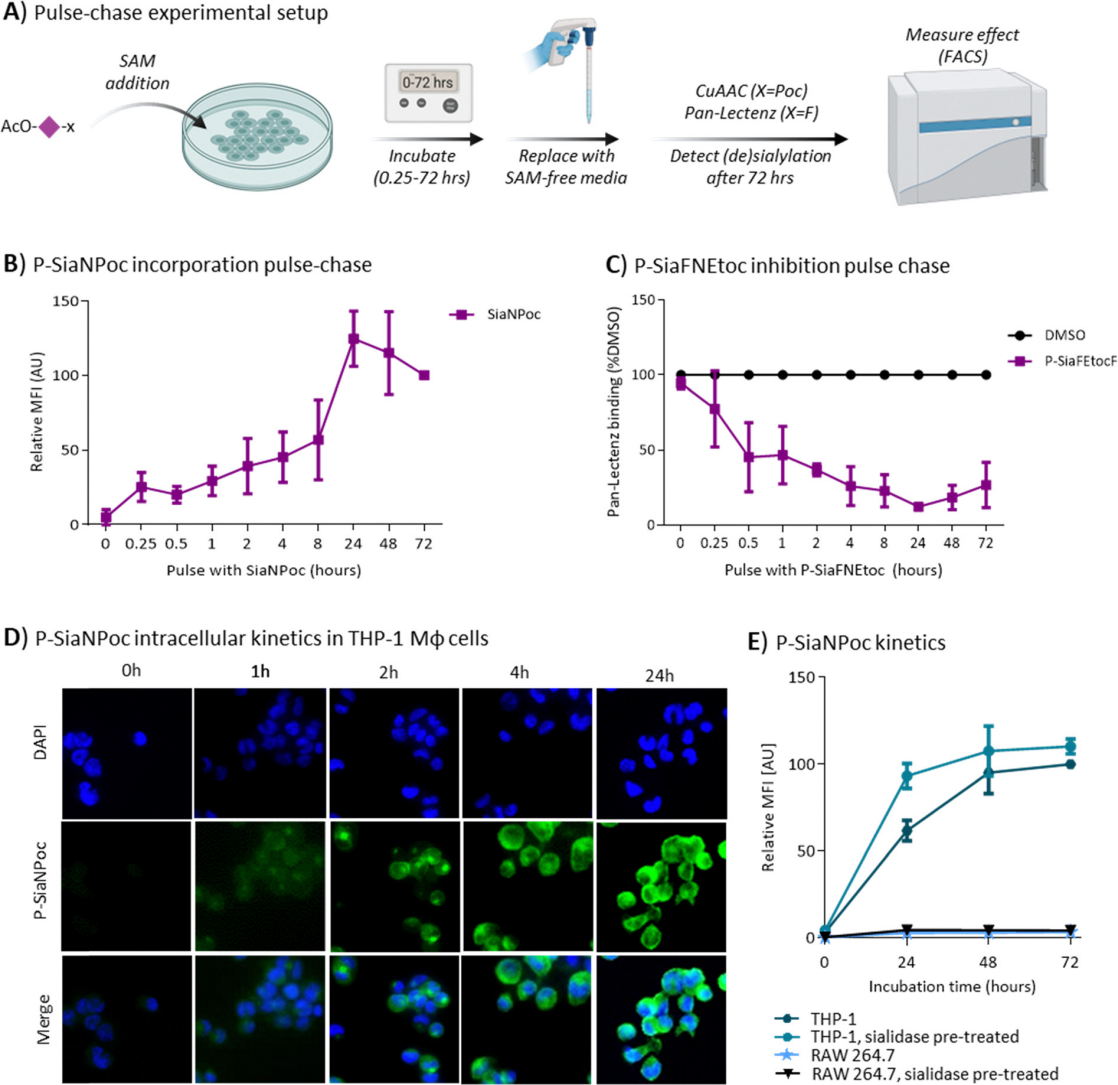
Effect speed of SAMs in THP-1 and RAW 264.7 cells. (A) Schematic representation of the pulse–chase experiment. (B) and (C). THP-1 cells were cultured with either 100 μM P-SiaNPoc (B) or 100 μM P-SiaFNEtoc (C) for the indicated time points, after which the SAM-containing medium was replaced with new culture medium without SAMs. After a total of 72 hours, P-SiaNPoc incorporation and effects of P-SiaFNEtoc on surface sialoglycans was measured by flow cytometry using CuAAC and Pan-Lectenz staining, respectively. Graphs shows P-SiaNPoc incorporation as average relative mean fluorescence (MFI) values ±SD normalized to signal from DMSO-treated cells (B) and average Pan-Lectenz binding ±SD normalized to control DMSO-treated cells (C) (*n* = 3). (D) Representative 40× fluorescence microscopy images show SiaNPoc-labeled sialoglycans in THP-1 Mφ cells. Cells were incubated for 0, 1, 4 and 24 hours with P-SiaNPoc, permeabilized and Poc-containing sialoglycans are visualized by reaction to a fluorophore. Nuclei were stained with DAPI. (E) Effect of sialidase treatment on P-SiaNPoc incorporation. THP-1 and RAW 264.7 cells were treated with or without sialidase for 45 minutes, after which the cells were incubated with 100 μM P-SiaNPoc for 24, 48, or 72 hours. P-SiaNPoc incorporation was measured after CuAAC to a fluorophore using flow cytometry. Graph shows the average relative MFI values ±SD normalized to control DMSO-treated cells (*n* = 3). Elements of panel A were created using Biorender.

The differences in activity of the SAMs and especially the low responsiveness macrophage(-like) cell lines prompted us to investigate the kinetics of P-SiaNPoc and P-SiaFNEtoc utilization. The SAMs are peracetylated to enable passive diffusion over the cell membrane. We performed pulse–chase experiments to investigate how long it takes until the SAMs are sufficiently taken up by cells. To this end, THP-1 cells and RAW 264.7 cells were incubated for different periods of time with either P-SiaNPoc or P-SiaFNEtoc followed by removal of the medium containing the SAM by washing with fresh medium. Next, the cells were cultured for 72 hours to allow for metabolic processing and sialoglycan turnover (Fig. 2A).^29^ P-SiaNPoc incorporation was measured using a bioorthogonal reaction to introduce a fluorophore and inhibition of sialylation by P-SiaFNEtoc was measured with Pan-Lectenz. Poc groups at the cell surface were detectable after a pulse of 15 minutes with P-SiaNPoc and the signal reached a maximum after 24 hours of incubation with P-SiaNPoc (Fig. 2B). Similarly, a 15-minute pulse with P-SiaFNEtoc significantly reduced sialic acid capping in THP-1 cells (Fig. 2C) and the reduction in sialylation gradually increased with longer pulse times and reached maximum inhibition around 4–8 hours of exposure to P-SiaFNEtoc. These experiments indicate that THP-1 cells take up effective concentrations of the SAMs within hours and that their effects are detectable after several days. Maximum inhibition of sialylation with P-SiaFNEtoc was achieved with lower pulse time compared to the maximum incorporation of P-SiaNPoc. This difference may be explained by the different mode of action of the two compounds. P-SiaFNEtoc leads to a metabolic blockade which is associated with a lower threshold concentration which is expected to remain rather stable as no efflux mechanism of CMP-Sia is known and dilution only takes place as a result of cell proliferation. In contrast, P-SiaNPoc results in a metabolically active substrate that needs to compete with endogenous sialic acid and is consumed. Hence, it may require higher concentrations of P-SiaNPoc to observe its incorporation into sialoglycans. Next, we investigated the intracellular kinetics of SiaNPoc processing. To this end, THP-1 Mφ cells were treated with 100 μM P-SiaNPoc for various time points and intracellular and membrane sialoglycans were fluorescently labelled with a CuAAC reaction to incorporate a fluorescent label followed by detection by fluorescence microscopy (Fig. 2D). After two hours of treatment, SiaNPoc-derived signal was visible in defined intracellular compartments, most likely the Golgi apparatus where sialoglycans are assembled. We have previously shown that P-SiaNPoc indeed labels the *trans*-Golgi.^45^ Further analysis of THP-1 cells with flow cytometry showed that the presence of Poc groups on the cell surface reaches a maximum after 48 hours of incubation (Fig. 2E). Finally, we investigated how the extracellular sialylation status of the cell influences sialoglycan metabolism and turnover. We treated cells (THP-1 or RAW 264.7) with sialidase to remove cell surface sialic acids before P-SiaNPoc incubation. In case of THP-1 cells, the incorporation of P-SiaNPoc after 24 hours was enhanced (Fig. 2E). This suggests that sialidase treatment increases the turnover, or possibly the re-sialylation of surface sialoglycans, but this effect requires further investigation. In contrast, RAW 264.7 cells showed no incorporation of P-SiaNPoc after 72 hours and sialidase treatment did not change this (Fig. 2E). This was supported by analysis of RAW 264.7 cells with fluorescent microscopy after incubation with P-SiaNPoc where no intra- or extracellular signal was detectable (Fig. S4). Similar experiments using metabolic inhibitor P-SiaFNEtoc took approximately 72 hours to reach a maximum. Pre-treatment with sialidase followed by addition of P-SiaFNEtoc to the culture medium effectively depleted cell surface sialic acids immediately (Fig. S5). This suggests that combined sialidase and P-SiaFNEtoc treatment can be used to directly remove sialic acids and lasts for a prolonged time, except for RAW 264.7 cells. Notably, cell surface sialylation after sialidase treatment fully restored within 24 hours in THP-1, HEK293, and in RAW 264.7 cells (Fig. S5 and S6) indicating that both cells actively biosynthesize sialic acids. Hence, their differential utilization of SAMs is likely the result of differences in uptake or processing by esterases needed to afford the respective metabolic precursors.

### Esterase activity is a bottleneck for activity of SAMs

**Fig. 3:**
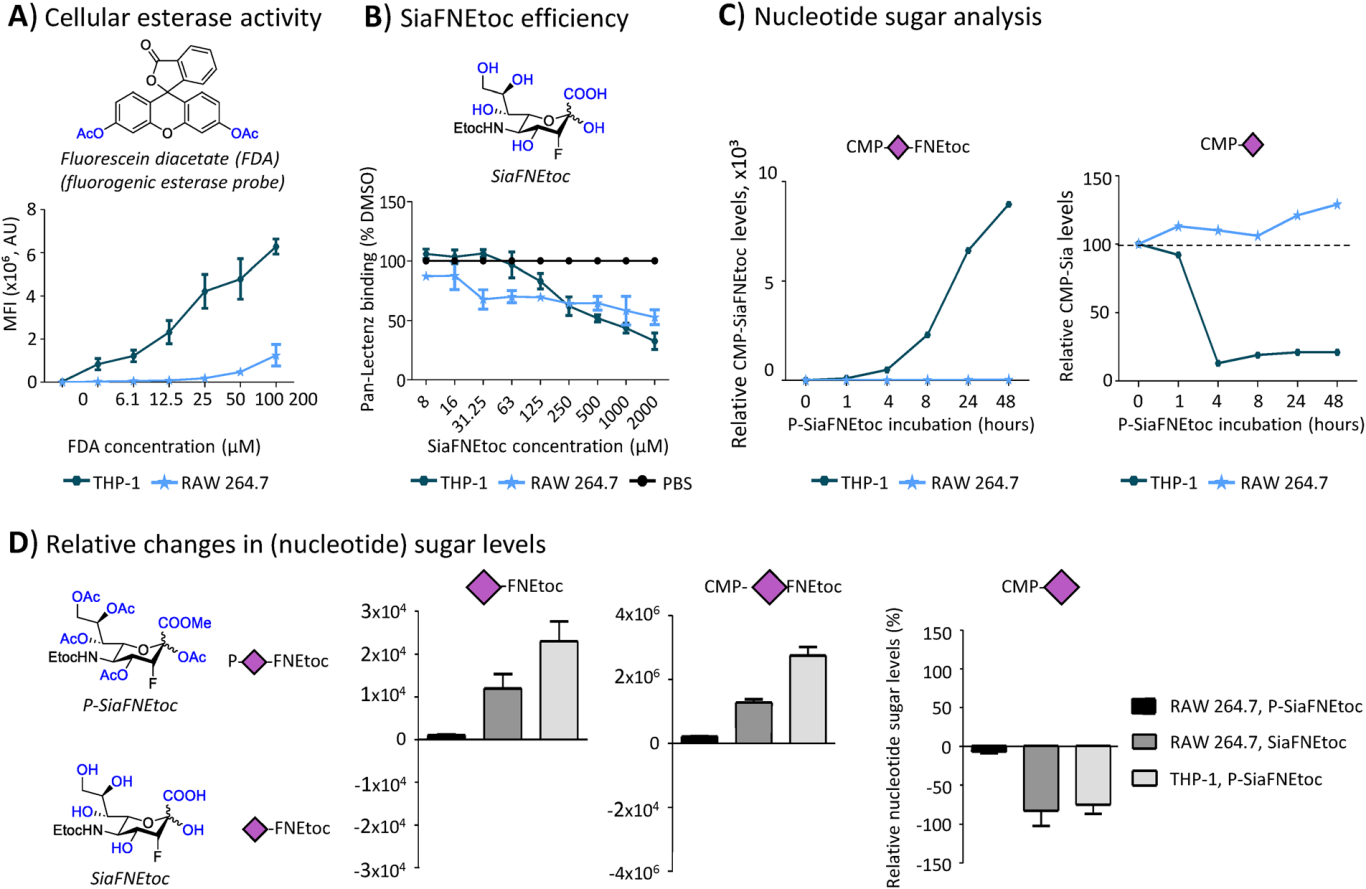
Utilization and CMP-activation of protected and unprotected SAMs. (A) THP-1 and RAW 264.7 cells were incubated with increasing concentrations of fluorescein diacetate (FDA) for 10 minutes and fluorescence was measured by flow cytometry. Graph shows the average mean fluorescence intensities (MFI) ±SEM (*n* = 3). (B) THP-1 and RAW 264.7 cells were incubated with a concentration range of unprotected SiaFNEtoc for 72 h or PBS control followed by detection of surface sialoglycans with Pan-Lectenz and flow cytometry. The graph shows the average Pan-Lectenz binding ±SEM normalized to control PBS-treated cells (*n* = 3). (C) Nucleotide sugar analysis of THP-1 and RAW 264.7 cells treated for the indicated time points with P-SiaFNEtoc or DMSO control. Representative graphs of two independent experiments show relative CMP-SiaFNEtoc levels (left) and relative CMP-Neu5Ac levels (right) normalized to DMSO-treated cells. (D) THP-1 and RAW 264.7 cells were treated for 24 hours with 100 μM P-SiaFNEtoc, 1 mM SiaFNEtoc, or DMSO control and nucleotide sugar levels were measured. Representative structures of P-SiaFNEtoc and SiaFNEtoc are shown (left). Bar diagrams show relative levels of intracellular levels of CMP-Neu5Ac (left), CMP-SiaFNEtoc (middle), and SiaFNEtoc (right) normalized to respective DMSO or PBS controls. Average values ±SD of three independent experiments are shown.

RAW 264.7 cells showed no incorporation of P-SiaNPoc and were also insensitive to inhibition with P-SiaFNEtoc. This suggests that the cell entry or intracellular deprotection and nucleotide sugar activation may not be effective. First the ability of RAW 264.7 cells to de-esterify peracetylated prodrugs was investigated. Since it is unknown if a specific esterase or multiple esterases deprotect SAMs, we probed the overall activity of cytoplasmic esterases with the fluorescently quenched esterase probe fluorescein diacetate (FDA). FDA is cell-permeable and becomes fluorescent once its two acetyl groups are removed by intracellular esterases. THP-1 monocytes and RAW 264.7 cells were incubated with increasing concentrations of FDA and fluorescence was analyzed by flow cytometry (Fig. 3A). THP-1 cells produced a fluorescent signal in a dose-dependent manner, while RAW 264.7 cells showed only a minor fluorescence signal at the highest concentrations (Fig. 3A). This suggests that this macrophage cell line has low esterase activity and that this could be the reason why the peracetylated SAMs were ineffective in RAW 264.7 cells. A similar trend was observed for THP-1 monocytes and macrophages (Fig. S7). To further explore this hypothesis, we produced deprotected SiaFNEtoc that does not require deacetylation and cultured THP-1 cells and RAW 264.7 cells for 72 hours with increasing concentrations. Both cell lines showed reduced sialylation and an approximate 50% reduction at concentrations >1 mM (Fig. 3B). This high concentration is needed because this sialic acid is poorly cell-permeable and likely enters cells *via* pinocytosis as has been shown for other unprotected SAMs.^20,21^ This finding strongly supports our hypothesis that a lack of esterase activity is the factor limiting SAM activity in RAW 264.7 cells. To ensure that the follow up step, activation of the SAMs with CMP by CMAS, is functional, we measured nucleotide sugar levels in THP-1 cells and RAW 264.7 cells treated with SAMs using LC-MS.^46,47^ After addition of P-SiaFNEtoc, CMP-SiaFNEtoc was detectable in THP-1 cells after 4 hours of incubation and further increased in time (Fig. 3C). Accordingly, endogenous CMP-Neu5Ac levels were strongly reduced within 4 hours of incubation (Fig. 3C). These data are in line with our pulse–chase experiments showing that 4 hours incubation is sufficient to achieve effective inhibition of sialylation (Fig. 2C). In RAW 264.7 cells, no CMP-SiaFNEtoc was formed even after 24-hour incubation with P-SiaFNEtoc and endogenous CMP-Neu5Ac levels remained stable and even slightly increased (Fig. 3C).

Using the LC-MS analysis, we could directly measure the levels of unprotected SiaFNEtoc in both RAW 264.7 and THP-1 cells following 24 hours of incubation (Fig. 3D). Much reduced SiaFNEtoc levels were observed when using P-SiaFNEtoc as the precursor in RAW 267.7 cells suggesting that inefficient esterase processing is likely causing this difference. This is further supported by the CMP-SiaFNEtoc levels which showed a similar trend. Finally, the consequence of differential metabolic processing of P-SiaFNEtoc and SiaFNEtoc in RAW 264.7 cells is reflected by the endogenous CMP-Neu5Ac levels which were strongly reduced in the latter case but remained unaffected by P-SiaFNEtoc treatment. Finally, the levels of other nucleotide sugars remained unaffected by the treatment (Fig. S8). Altogether, these data show that cellular esterase activity forms a bottleneck for the activity of peracetylated SAMs, consistent with observations made by others.^24,42^ While peracetylated SAMs work efficiently in most mammalian cell lines, RAW 264.7 cells and human macrophages derived from blood monocytes or THP-1 cells show no or intermediate utilization, respectively. Publicly available RNA-sequencing data showed that RAW 264.7 cells express genes involved in sialoglycan biosynthesis, but have low expression levels of cytosolic esterases that may act on the SAMs compared to THP-1 cells (Table S1).^48,49^ Differentiation of THP-1 cells into macrophages was shown to slightly reduce esterase expression and may partly explain why the THP-1 Mφ are less sensitive to SAMs.^49^ Future research is needed to identify single or multiple esterases that mediate the deprotection of peracetylated SAMs.

## Conclusions

SAMs are versatile tools to probe and perturb sialylation in living cells and hold potential therapeutic value for application in oncology and other areas. Many aspects regarding the metabolic fate of SAMs including cellular uptake, activation, and turnover are not understood in detail. This information is important to understand the cell type-specific utilization and metabolism of SAMs and to maximize their (targeted) application in different cell types and tissues. The peracetylated SAMs tested in this study potently inhibited sialic acid capping and were incorporated in several mammalian cell lines. Differences in their potency between cell lines were found and we identified esterase activity as bottleneck for the activity of peracetylated SAMs. These findings provide relevant considerations for the application of SAMs in mammalian cells and instruct future SAM development.

## Author contributions

E. A. V., D. L. A. H. H., D. J. L., C. B., T. J. B. conceptualized the experimental plan. E. A. V., D. L. A. H. H., M. R., O. O., V. P., M. C., C. M., S. J. M. and M. J. performed the experiments. E. A. V., D. L. A. H. H., C. B. and T. J. B. visualized and wrote the original draft. All authors contributed to review & editing of the manuscript and SI.

## Conflicts of interest

C. B. and T. J. B. are co-founders of GlycoTherapeutics BV and hold ownership in the company. T. J. B. and S. J. M are co-founders of Synvenio B.V. and hold ownership in the company.

## Supplementary Material

CB-OLF-D5CB00193E-s001

## Data Availability

The data supporting this article have been included as part of the supplementary information (SI). Supplementary information: additional references from the SI contain information about the materials and methods. The authors have cited additional references within the SI.^46–53^ See DOI: https://doi.org/10.1039/d5cb00193e.
