# Supplementary material for: Investigating the utilization mechanism and kinetics of sialic acid mimetics in mammalian cell lines
Source: RSC Chem Biol. 2025 Oct 29;7(1):144–52. doi: 10.1039/d5cb00193e (PMC12580913; doi:10.1039/d5cb00193e)
Supplement: CB-007-D5CB00193E-s001 [file CB-007-D5CB00193E-s001.pdf]

Supplementary Information (SI) for RSC Chemical Biology.
This journal is © The Royal Society of Chemistry 2025

Supporting Information

Investigating the Utilization Mechanism and Kinetics of Sialic Acid Mimetics in Mammalian Cell Lines

Eline A. Visser,[a] Daniël L.A.H Hornikx,[b] Moritz Rahm,[c] Özden Öztürk,[a] Venetia Psomiadou,[a] Matteo Calzari,[a] Celine Mennen,[a] Sam J. Moons,[d] Martin Jaeger,[e] Dirk J. Lefeber,[c,f] Christian Büll*[b] and Thomas J. Boltje*[a]

[a] Department of Synthetic Organic Chemistry, Institute for Molecules and Materials, Radboud University Nijmegen, Toernooiveld 1, 6525 ED Nijmegen, The Netherlands
[b] Department of Biomolecular Chemistry, Institute for Molecules and Materials, Radboud University Nijmegen, Heyendaalseweg 135, 6525 AJ Nijmegen, The Netherlands
[c] Department of Laboratory Medicine, Translational Metabolic Laboratory, Radboud University Medical Center, Geert Grooteplein Zuid 10, 6525 GA, Nijmegen, The Netherlands
[d] Synvenio B.V., Toernooiveld 1, 6525 ED Nijmegen, The Netherlands
[e] Department of Experimental Internal Medicine, Radboud Institute for Medical Innovations, Radboud University Medical Center, Geert Grooteplein Zuid 10, 6525 GA, Nijmegen, The Netherlands
[f] Department of Neurology, Donders Institute for Brain, Cognition, and Behavior, Radboud University Medical Center, Geert Grooteplein Zuid 10, 6525 GA, Nijmegen, The Netherlands
* Corresponding authors: christian.bull@ru.nl and thomas.boltje@ru.nl

Table of Contents

Supplementary Tables and Figures…………………………..……………………………...............2

Materials and Methods..10

References……………………….…………………………………………………….……………….12

Supplementary Tables and Figures

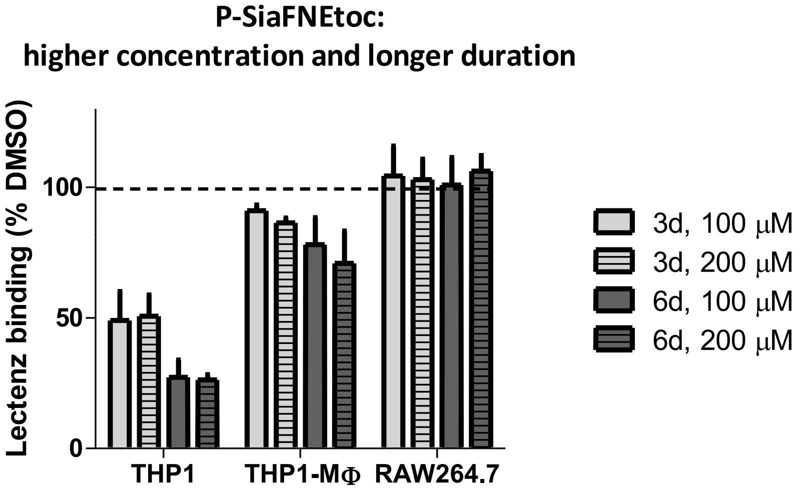

Figure S1. THP1, THP1-Mφ, and RAW264.7 cells were incubated with an indicated concentration of P-SiaFNEtoc or a DMSO control for 3 or 6 days, after which Lectenz binding levels were measured via Flow cytometry. Treated values are relative to the DMSO control, and data is depicted with the SD.

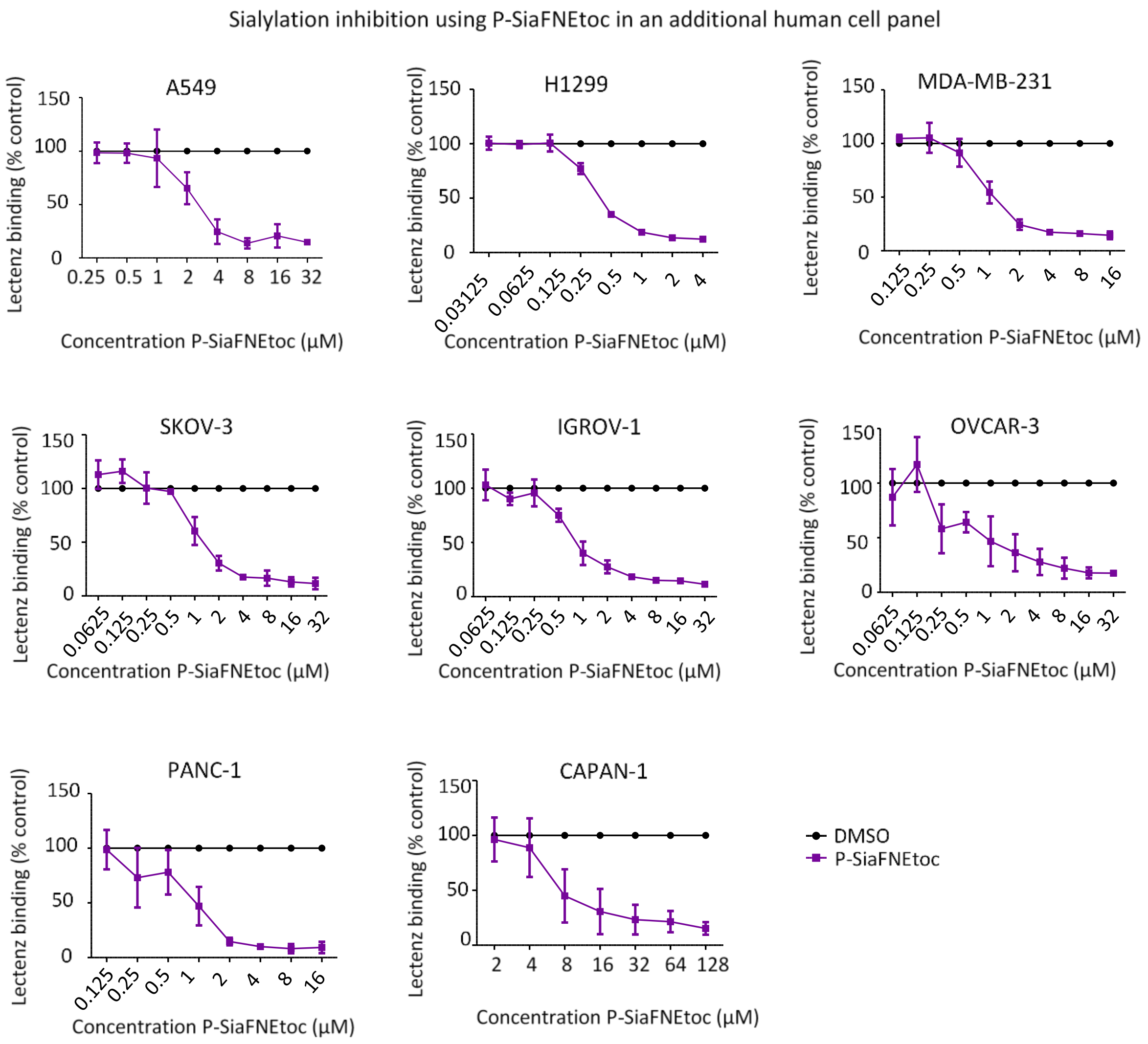

Figure S2. A549, H1299, MDA-MB-231, SKOV-3, IGROV-1, OVCAR-3, PANC-1 and CAPAN-1 cells were incubated with an indicated concentration range of P-SiaFNEtoc or a DMSO control for 3 days, after which Lectenz binding levels were measured via Flow cytometry. Treated values are relative to the DMSO control, and data is depicted with the SD.

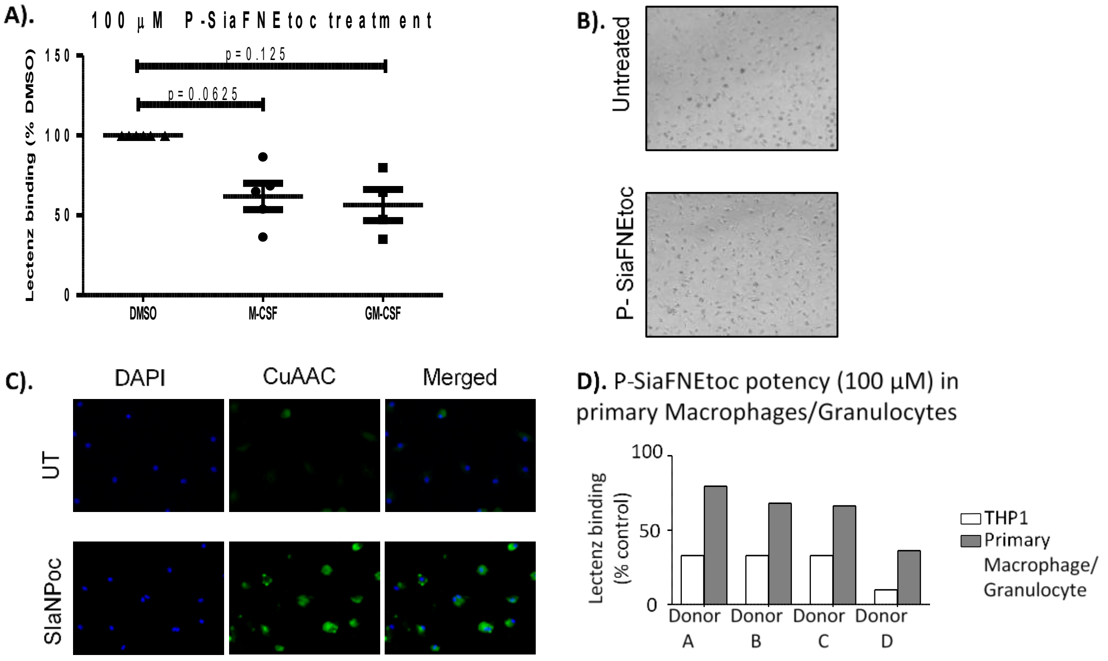

Figure S3. Activity of SAMs in PBMC-derived macrophages. **A**). Relative quantification of sialic acid levels on PBMC-derived macrophages after 72h incubation using 100 µM P-SiaNFEtoc. Data is presented as mean fluorescent intensity of *n*=5 (M-CSF) and *n*=4 (GM-CSF) differentiated macrophages. Data is normalised against DMSO-treated M-CSF replicate. Statistics are determined using a Wilcoxon matched-pairs signed rank t-test. **B**). Microscopic image of DMSO (top) and P-SiaFNEtoc (bottom) treated M-CSF stimulated PBMCs. **C**). Fluorescent image of M-CSF differentiated PBMCs, treated with 100 µM P-SiaNPoc for 48h. Cells were fixed using 4% PFA and blocked in 5% (w/v) BSA in 1x PBS. SiaNPoc-incorporated sialoglycans were functionalised using CuAAC-reaction containing 50 µM N_3-PEG_3-biotin and visualised using 1 µg/ml AlexaFluor488-conjugated streptavidin. Nuclei were visualised using 1µg/ml DAPI. Images were taken on theEVOSM5000 using the 40x objective. **D**). PBMC's differentiated with M-CSF or GM-CSF and THP1 cells were treated with 100 µM P-SiaFNEtoc or a DMSO control for 3 days, after which Pan-Lectenz binding was measured using flow cytometry. Treated values are relative to the DMSO control.

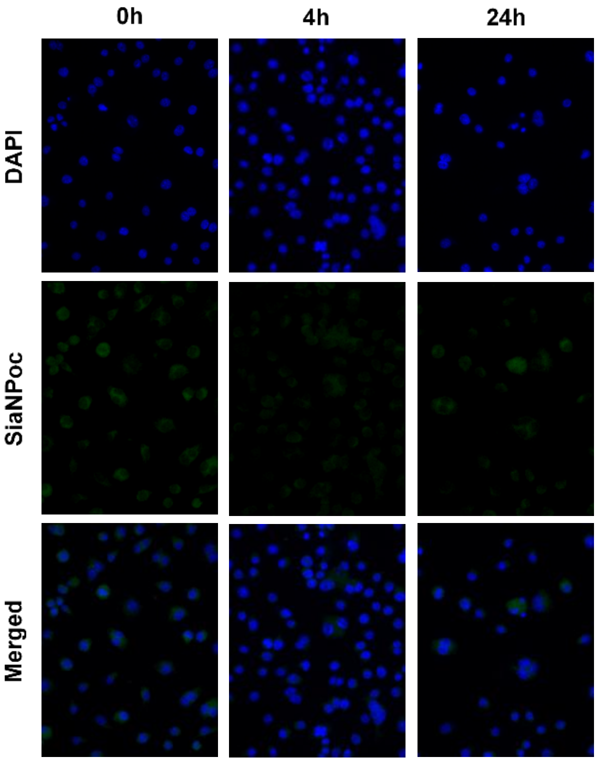

Figure S4. Chasing SiaNPoc-incorporation inside RAW264.7 cells. Immunofluorescence microscopy detection of SiaNPoc-labeled sialoglycans. Cells were incubated for 0, 4 and 24 hours with P-SiaNPoc, and fixed using PFA. SiaNPoc-incorporated sialoglycans were functionalized using CuAAC-reaction with a N_3-PEG3-biotin and visualized using AlexaFluor488-conjugated Streptavidin. Nuclei were stained using DAPI, prior to mounting coverslips to a histology slide. Images were taken using the EVOS M5000 Imaging system using the 40x objective.

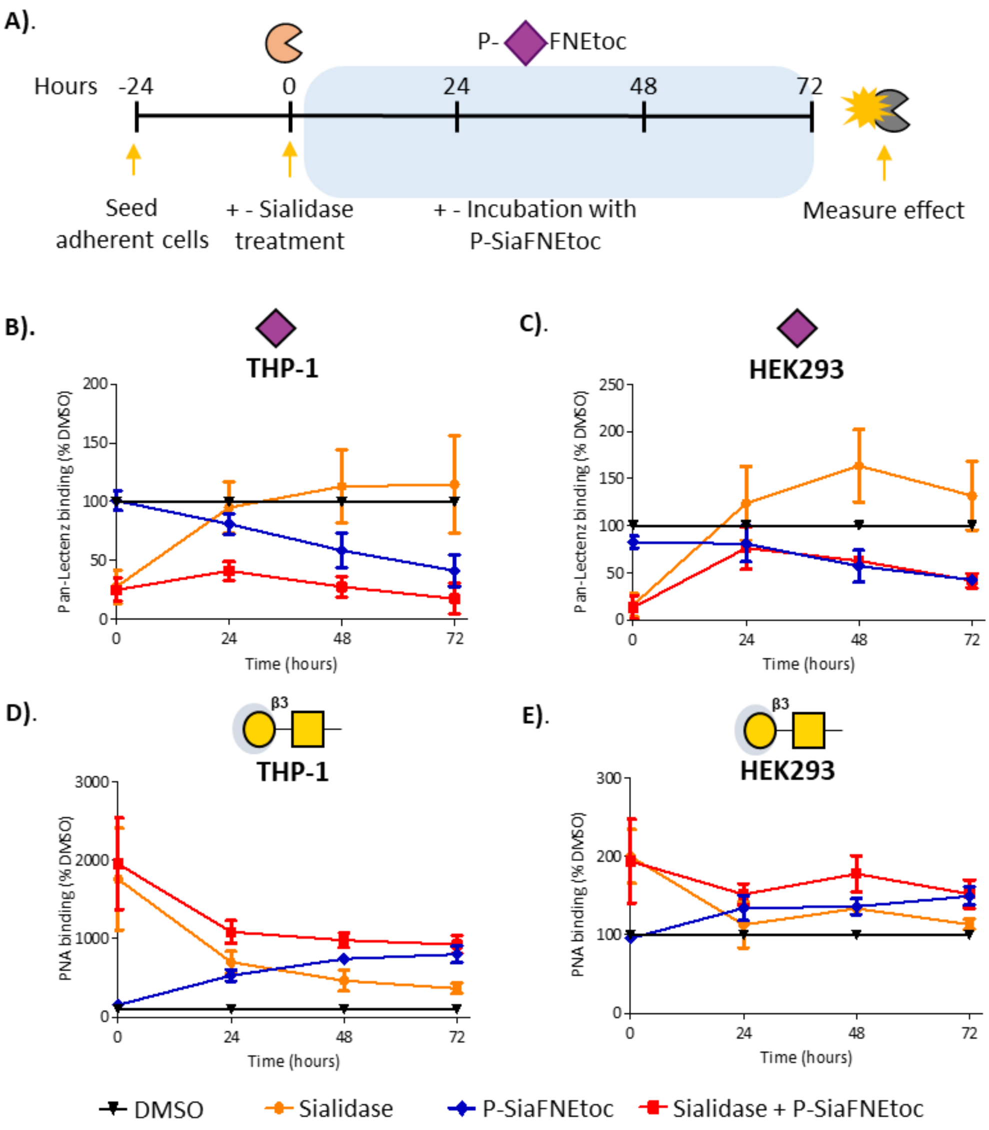

Figure S5. Combined enzymatic and metabolic desialylation. **A**). Schematic representation of experimental set-up. THP-1 and HEK293 were treated with 100 mU/ml *C. perfringens* sialidase for 45 minutes to remove extracellular sialic acids, after which sialylation levels are followed 0, 24, 48 and 72 hours after treatment using Pan-Lectenz, and the underlying glycan structures uncovered by Sia removal using PNA lectin staining. In addition, cells were either treated or not with C. Perfringens sialidase for 45 minutes, after which the cells were incubated with 50 µM P-SiaFNEtoc. After the indicated time points, Lectenz binding and PNA binding was measured on the cell surface measured by flow cytometry. **B-C**). The relative binding values of PAN-Lectenz binding in HEK293 cells (**B**) and THP-1 cells (**C**). Data is depicted as normalized mean fluorescent intensity ± SD normalized to the DMSO treated controls. **D-E**). The relative binding values of PNA-lectin binding, in HEK293 cells (**D**) and THP-1 cells (**E**). Data is depicted as normalized mean fluorescent intensity ± SD normalized to the DMSO treated controls.

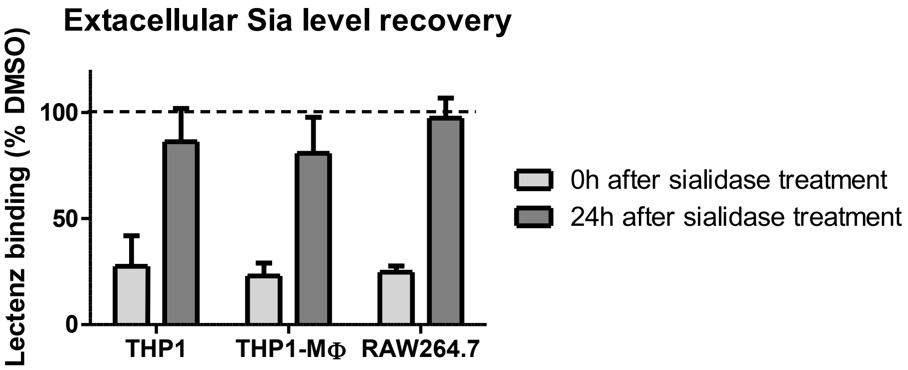

Figure S6. Investigating sialoglycan turnover after sialidase treatment. THP1, THP-1 Mφ and RAW264.7 cells were treated with 100 mU/ml *C. perfringens* sialidase for 45 minutes to remove extracellular sialic acids, after which sialylation levels are followed 0 and 24 hours after treatment using pan-specific Lectenz and flow cytometry. Data is depicted as normalized mean fluorescent intensity±SD normalized to the DMSO treated controls, n=3.

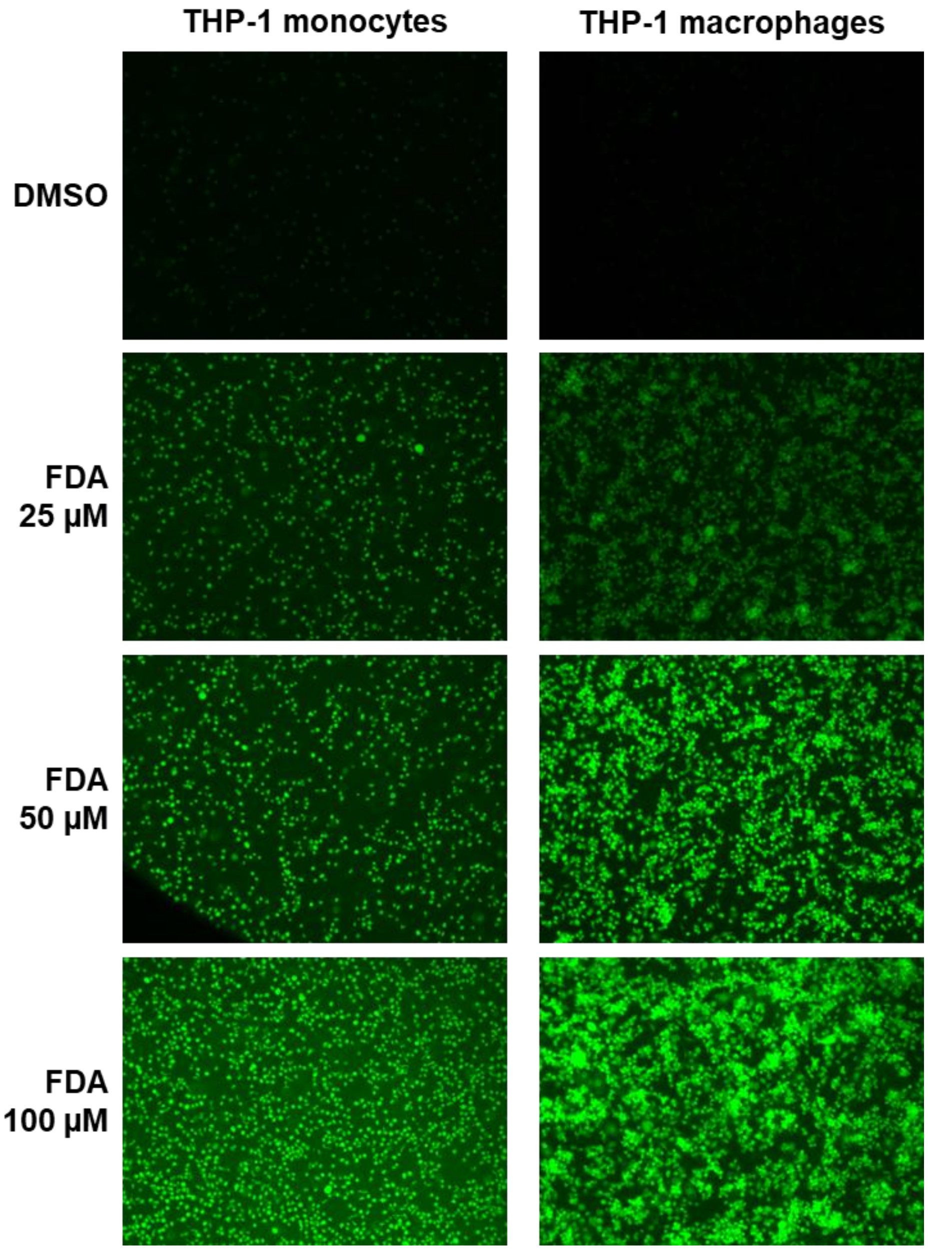

Figure S7. Utilization of FDA by THP-1 monocytes and macrophages. Representative fluorescence microscopy images show THP-1 monocytes (suspension) and macrophages (attached) after 20 minutes incubation with different concentrations of FDA.

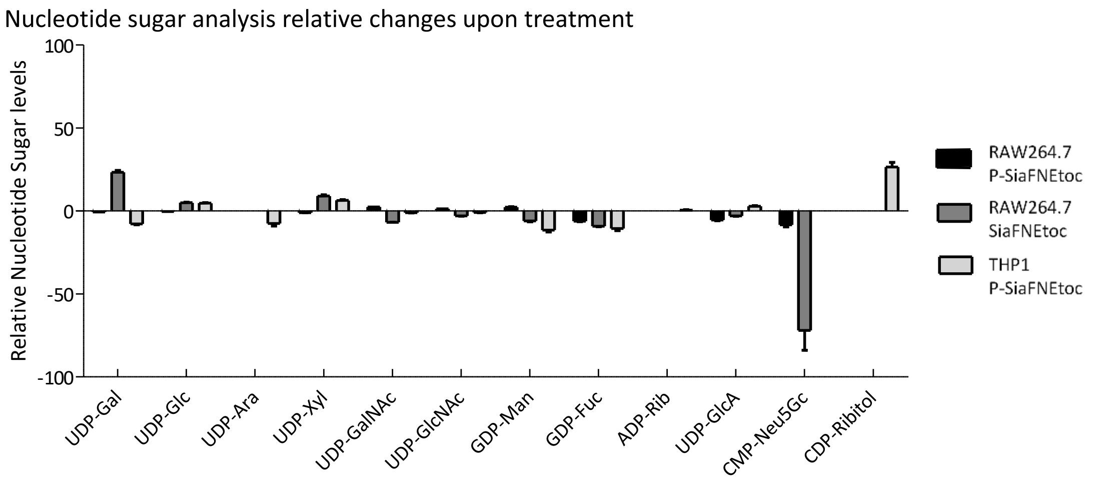

Figure S8. Nucleotide sugar levels measured via mass spectrometry of RAW264.7 and THP1 cells treated for 24h with P-SiaFNEtoc, SiaFNEtoc or their respective DMSO or PBS controls. Levels are normalized to the DMSO or PBS controls. Data is depicted with SD.

	THP-1	THP-1 Mϕ	RAW 264.7
		**Sialyltransferases**	
ST3GAL1	0	0.050142	3.5
ST3GAL2	1.868	1.83574	3.2
ST3GAL3	0	0	6.7
ST3GAL4	0	0.320845	10.2
ST3GAL6	4.68493	12.02668	46.7
ST6GAL1	0	0.229348	40.9
ST6GAL2	0	0	8.4
ST6GALNAC1	0	0	0
ST6GALNAC2	1.40202	1.033845	0
ST6GALNAC3	0.053479	0.048361	0
ST6GALNAC4	0.195006	4.36662	0
ST6GALNAC5	0	0	29.5
ST6GALNAC6	0	0.000103	0
ST8SIA1	0.940301	0.834225	0
ST8SIA2	0	0	0
ST8SIA3	0	0	0
ST8SIA4	2.228875	21.53365	25.2
ST8SIA5	0.432534	13.80235	0
ST8SIA6	0.078	0.020436	0
		**Reference genes**	
GAPDH	1.880395	5.078615	1172.5
B2M	198.0705	1360.58	834.8
ACTB	604.837	477.934	1657.6

	THP-1	THP-1 Mϕ	RAW 264.7
		**Esterases**	
ACHE	0	0	0.2
CES1	18.14757	6.215705	0
CES2	3.32555	7.327695	0
CES3	1.458735	0.144428	0
CES4A	1.48378	1.308655	0
CES5A	0	0	0
COLQ	0	0.001325	0
ESD	9.154105	8.76292	79.3
ACOT11	0.249145	0.790895	0.2
CNP	0.808528	2.88913	4.1
PAFAH1B2	7.226355	8.849885	0
PAFAH1B3	0	0.471703	0
CEL	1.645385	0.780875	0

Table S1. Heat-map showing RNA-sequencing data for sialyltransferases, esterases, and reference genes in THP-1, THP-1 Mφ, and RAW 264.7. Data were reused from: [1]

Materials and Methods

Many of the methods used are also more elaborately explained in STAR protocols.[2] P-SiaFNEtoc,[3] SiaFNEtoc[4] and P-SiaNPoc[5] were synthesized as described previously.

Cell culture

THP-1 cells (TIB-202, ATCC) were cultured in RPMI-1640 medium containing 2mM Glutamine and 25mM HEPES (GibcoTM, Life Technologies), supplemented with 10% v/v heat-inactivated fetal bovine serum (FBS) (GibcoTM, Life Technologies) and 1X antibiotic-antimycotic solution (100 units/mL of penicillin, 100 µg/mL of streptomycin, and 0.25 µg/mL Fungizone) (GibcoTM, Life Technologies). To obtain THP-1 Mφ cells, THP-1 cells were treated with 12ng/mL PMA (Phorbol 12-myristate 13-acetate (SC-3576, Santa Cruz Biotechnology) for 24 hours, after which the media with PMA was removed from the now adherent cells and refreshed with new media. THP-1 cells were passaged until a passage number of 25.

RAW264.7, MC38 (ATCC), HEK293 (ATCC), and SH-SY5Y (ATCC) cells were cultured in DMEM medium (GibcoTM, Life Technologies), supplemented with 10% v/v heat-inactivated fetal bovine serum (FBS) (GibcoTM, Life Technologies), 1X antibiotic-antimycotic solution (100 units/mL of penicillin, 100 µg/mL of streptomycin, and 0.25 µg/mL Fungizone) (GibcoTM, Life Technologies). All cells were cultured at 37°C, 5% CO_2 in a humidified incubator, and passaged (for the adherent cells with trypsin-EDTA 0.5% (Gibco, ThermoFisherScientific) every 3-4 days. Cells were passaged until a maximum passage number of 30–35 after thawing.

Sialidase treatment

For sialidase treatment, adherent cells were seeded on 24-wells plates (Corning) and allowed to adhere for 24h (RAW264.7 and HEK293 100,000 cells/well). Suspension cell line THP-1 grew in 96-wells plates (THP-1 suspension cell line; 80,000-160,000 cells per well) Cells were washed with PBS (HEK293 cells were first removed from the plate using sterile PBS (Gibco, ThermoFisherScientific), after which they were collected by centrifugation (300×g, 5 minutes at 4°C) and incubated with 100mU *C. perfringens* sialidase (Sigma-Aldrich, N2876) in serum free media for 45 minutes at 37°C. After this, cells were washed in PBS again and resuspended in normal serum containing media, either with or without SAMs. RAW264.7 cells could be kept adherent during this procedure.

Cell culture SAM treatment for Potency Curves

Adherent cells were seeded on 48-wells plates and allowed to adhere for 24 hours (THP-1 Mφ: 500,000-1,000,000 cells/well including 12ng/mL PMA for differentiation, RAW264.7, HEK293 and MC38 40.000-50,000 cells/well, SH-SY5Y 200,000 cells/well) (Corning). Suspension cell line THP-1 grew in 96-wells plates (THP-1 suspension cell line; 80,000 cells per well) (ThermoFisherScientific). After 24 hours, media was removed from cells and replaced with media containing different concentrations (0–128 µM, two fold dilutions) of SAMs or a DMSO (Sigma-Aldrich) control. Cells incubated for three days at 37°C and 5% CO2 in a humidified incubator, after which cells were harvested, stained and the fluorescence was measured. For the onset experiment, the cells incubated with SAMs for a different amount of time, as indicated, either with or without sialidase treatment prior to addition of P-SiaFNEtoc.

CuAAC-reaction for cell surface labeling measured via Flow Cytometry (incorporative SAMs)

In order to stain the cells for flow cytometry, adherent cells were harvested by trypsinization (3 minutes max. and de-activated using serum containing media), scraping (RAW264.7 cells) or EDTA-detachment (PBMCs) and transferred to a 96-wells V-bottom plate (Thermo Scientific). Cells pelleted by centrifugation (2000rpm at 4°C) and were washed with 100 µL PBS. The cells were then resuspended in 95 µL of reaction buffer (250 µM $CuSO_4$ (Sigma-Aldrich), 200 µM L-histidine (ThermoFisherScientific), 100 µM of azide-PEG3-biotin conjugate (Sigma-Aldrich) in PBS), and 5 µL of a freshly made solution of sodium ascorbate (10 mM in PBS, final concentration of 500 µM) (Sigma-Aldrich) was added, after which cells were incubated at 37 °C for 20 min. Cells were washed three times with 100 µL PBS and subsequently incubated with 40 µL 0.8 µg/mL streptavidin–PE conjugate (Invitrogen, eBioscience) in PBA (PBS containing 1% v/v FBS and 0.02% w/w sodium azide) for 10 min at 4 °C. Cells were washed again three times using 100 µL of PBS, resuspended in PBA, and fluorescence was measured with a flow cytometer (Beckman & Dickinson FACSCalibur). Each replicate for each condition contained n≥10,000 gated events, and the protocol was repeated at separate days using separate passage numbers until n≥3 was reached. Data was processed using FlowJo (FlowJo LLC), and GraphPad PRISM.

Lectenz and PNA staining via Flow cytometry (inhibitory SAMs)

In order to stain the cells for flow cytometry, adherent cells were harvested by trypsinization (3 minutes max. and de-activated using serum containing media) or scraping (RAW264.7 cells) and transferred to a 96-wells V-bottom plate (Thermo Scientific). Cells pelleted by centrifugation (2000rpm at 4°C) and were washed with 100 µL PBS. The cells were

then resuspended in either 40 µL of 5 µg/ml biotinylated PNA (Vector Laboratories inc.) or 2 µg/ml biotinylated Pan-specific Lectenz (LectenzBio) pre-complexed for 10 minutes with 0.8 µg/mL Streptavidin-PE (Invitrogen, eBioscience) in 1X carbo-free blocking buffer (Vector Laboratories Inc.) containing 1 mM $CaCl_2$ and 1 mM $MgCl_2$ and incubated at 4°C for 60 min. Cells were washed three times with 100 µL PBA (PBS containing 1% v/v FBS and 0.02% w/w sodium azide). Cells treated with biotinylated PNA subsequently incubated with 40 µL 0.8 µg/mL Streptavidin-PE in PBA for 10 min at 4°C. Cells were then washed again three times with 100 µL PBA, resuspended in PBA and fluorescence was measured with a flow cytometer (Beckman & Dickinson FACSCalibur, and Beckman & Coulter Cytoflex). Each replicate for each condition had n≥10,000 gated events, and the protocol was repeated at separate days using separate passage numbers until n≥3 was reached. Data was processed using FlowJo (FlowJo LLC), CytExpert and GraphPad PRISM. Percentage of lectin binding was obtained by normalizing the MFI values to the MFI values of the respective DMSO control.

Esterase activity measurement using fluorescent probes

$4x10^6$ RAW 264.7 cells were seeded in a 6-Well F-bottom plate and grown overnight. THP-1 monocytes were seeded at 500,000 cells/well on the day of the experiment in a 96 well V-bottom plate. Before use, RAW 264.7 cells were trypsinized and cells from one well were divided into 6 wells in a 96-well V-bottom plate. After centrifugation (2000 rmp at 4°C) and washing once with PBS, samples were treated with Fluorescein diacetate (FDA) esterase probe (F1303, Invitrogen) diluted in PBS at the indicated concentrations and incubated in a humidified 5%CO_2 incubator at 37°C for 10 minutes. Cells were then washed once with PBS, after which they were fixated with 1% Paraformaldehyde (PFA) (Sigma-Aldrich) in PBS at 37°C for 10 minutes. Afterwards, cells were washed with PBS and resuspended in PBS before measurement. Fluorescence was measured using a CytoFLEX Flow Cytometer. The protocol was repeated until n≥3 using cells at different passage numbers and measured on different days, and each replicate for each condition had n≥10,000 gated events.

Metabolite extraction and analysis

For the analysis of different time points of incubation with the P-SiaFNEtoc, 250.000 cells/well of THP-1 cells were plated per 6-wells well (ThermoFisher) and 32.000 cells/well in a 6 wells plate for RAW264.7 cells to ensure a total of around 1 million cells at time of harvesting. The next day, cells were treated with 100 µM P-SiaFNEtoc (or a similar volume of DMSO as a control) for the 48-hour time point. In the subsequent two days, both the 100 µM P-SiaFNEtoc and the DMSO were added at the appropriate times to ensure the right incubation times in a humidified 5% CO_2 incubator at 37°C before work-up.

For the next analysis we decided upon a 24-hour time point. For this, 400.000 THP-1 cells/well (6 wells plate) were seeded, and 140.000 RAW264.7 cells/well (6 wells plate) were seeded. The following day, cells were treated either with 100 µM P-SiaFNEtoc (or a similar volume of DMSO as a control) or 1mM SiaFNEtoc (or a similar volume of PBS as a control) in triplicate per condition for 24 hours of incubation in a humidified 5% CO_2 incubator at 37°C.After the incubation times with the compounds, medium was removed (either by vacuum (RAW264.7) or centrifuging after transfer of cells to eppendorfs (2000 rpm using a tabletop centrifuge) (THP-1) and the cells were washed twice with 1 (THP-1) or 2 (RAW264.7) mL 75 mM ammonium carbonate pH 7.4 buffer in MilliQ water. After removing the washing buffer, cells were snap-frozen in liquid nitrogen. Samples then were stored at -80°C until workup.

Samples were thawed at -20°C and incubated for 5 minutes in 1 mL of extraction buffer (THP-1) (1:2:2 MilliQ water: acetonitrile: methanol) or 1x 2 minutes in 700 µL and 1x 5 minutes in 700 µL extraction buffer (RAW264.7), after which those samples were pooled resulting in 1.4mL. Samples were then spun down at 13.000 rpm in a tabletop centrifuge and supernatant was transferred into 1.5 mL Eppendorf tubes and solvent was removed with a speedvac (Savant SC100, RVT 100 with an external vacuum pump).

The dried extract was redissolved in 20 µL of MilliQ water. Nucleotide sugar analysis was performed as previously described.[6] For the analysis of free Neu5Ac and SiaFNEtoc a second method was developed based on the method for nucleotide sugars. Gradient profile was adjusted for improved separation to denoted timepoints as follows (B in %): 0 min – 1.6 min: 5%, 2.5 min: 30%, 2.7 min - 4 min: 100%, 4.1 min – 6 min: 5%. Transitions for both sialic acids were determined from product ion scans and collision energies were optimized with 5 V precision. All LC-MS data files were processed in Skyline version 22.2. All transitions of the same precursor ion were summed and with the exception of CMP-SiaFNEtoc, CMP-Neu5Ac, SiaFNEtoc and Neu5Ac to avoid treatment dependent skewing. The average normalized peak area and coefficient of variation were calculated from the biological triplicates of each condition and cell line.

Immunofluorescence and microscopy - Subcellular localization

THP-1 cells were seeded onto 14 mm Ø glass coverslips stimulated with 15 ng/ml PMA for 16 hours. Cells were washed with 1x PBS and cultured in RPMI medium supplemented with 100 µM SiaNPoc in culture medium for a maximum of 24 hours. Stimulations under 8 hours were performed in serum-free culture medium (but containing pen/strep). After incorporation, cells were fixed using 1x PBS 4% (w/v) PFA and permeabilized using 0.1% (w/v) saponin (Sigma-Aldrich) in PBS. Blocking was performed using 1x PBS 5% (w/v) BSA 0.1% (w/v) saponin. All washing steps were performed using 1% (w/v) BSA 0.1% saponin in 1x PBS. CuAAC-reaction buffer (250 µM $CuSO_4$, 50 µM azide-PEG3-Biotin, activated with 500 µM sodium ascorbate) was made, supplemented with 0.1% saponin, and incubated for 45 minutes at 37°C. Nuclei were stained by incubation for 10 min with DAPI (Sigma-Aldrich). Coverslips were mounted using fluorescent mounting medium (DAKO). Images were made using the EVOS M5000 imaging system.

Statistics

To calculate the EC_{50} values, a non-linear fit curve of the inhibition was made using GraphPad PRISM using (log (inhibitor) vs. response – variable slope (four parameters) with the least squares fit, the top set at 100 and the bottom between 0 and 20 (due to residual Sia on the cell membrane). To determine the EC_{90}-values, a non-linear fit was made (log(Agonist) vs. response - Find ECanything) with F set at 10, the top at 100 and the bottom between 0 and 10. For the P-SiaNPoc, a non-linear fit curve was made using GraphPad PRISM using (log (inhibitor) vs. response – variable slope (four parameters) with the least squares fit and the bottom set at 0.

References

[1] aB. R. Sabari, Z. Tang, H. Huang, V. Yong-Gonzalez, H. Molina, H. E. Kong, L. Dai, M. Shimada, J. R. Cross, Y. Zhao, R. G. Roeder, C. D. Allis, *Molecular cell* **2015**, *58*, 203-215; bD. H. Phanstiel, K. Van Bortle, D. Spacek, G. T. Hess, M. S. Shamim, I. Machol, M. I. Love, E. L. Aiden, M. C. Bassik, M. P. Snyder, *Molecular cell* **2017**, *67*, 1037-1048.e1036.

[2] D. L. A. H. Hornikx, E. A. Visser, V. Psomiadou, C. Büll, T. J. Boltje, *STAR Protoc* **2023**, *4*, 102330.

[3] S. J. Moons, E. Rossing, M. A. C. H. Janssen, T. Heise, C. Büll, G. J. Adema, T. J. Boltje, *ACS Chem Biol* **2022**, *17*, 590-597.

[4] S. J. Moons, E. Rossing, J. J. A. Heming, M. A. C. H. Janssen, M. van Scherpenzeel, D. J. Lefeber, M. I. de Jonge, J. D. Langereis, T. J. Boltje, *Bioconjug Chem* **2021**, *32*, 1047-1051.

[5] S. J. Moons, A. D. Robertson, T. J. Boltje, *European Journal of Organic Chemistry* **2022**, *2022*, e202200659.

[6] a) M. Rahm, H. Kwast, H. Wessels, M. J. Noga, D. J. Lefeber, *Anal Bioanal Chem* **2024**, *416*, 3595-3604; b) M. van Scherpenzeel, F. Conte, C. Bull, A. Ashikov, E. Hermans, A. Willems, W. van Tol, E. Kragt, M. Noga, E. E. Moret, T. Heise, J. D. Langereis, E. Rossing, M. Zimmermann, M. E. Rubio-Gozalbo, M. I. de Jonge, G. J. Adema, N. Zamboni, T. Boltje, D. J. Lefeber, *Glycobiology* **2022**, *32*, 239-250.
